# Prelimbic cortical targets of ventromedial thalamic projections include inhibitory interneurons and corticostriatal pyramidal neurons in the rat

**DOI:** 10.1007/s00429-020-02109-3

**Published:** 2020-07-14

**Authors:** Bianca Sieveritz, Gordon W. Arbuthnott

**Affiliations:** Okinawa Institute of Science and Technology Graduate University, 1919-1 Tancha, Onna-son, Okinawa, 904-0495 Japan

**Keywords:** Ventromedial thalamus, Prelimbic cortex, Inhibitory interneurons, 5HT3aR, Corticostriatal neurons, RGS14

## Abstract

Ventromedial thalamic axons innervate cortical layer I and make contacts onto the apical dendritic tuft of pyramidal neurons. Optical stimulation of ventromedial thalamic axon terminals in prefrontal cortical areas in mouse brain slices evokes responses in corticocortical, corticothalamic and layer I inhibitory interneurons. Using anterograde tracing techniques and immunohistochemistry in male Sprague–Dawley rats, we provide anatomical evidence that ventromedial thalamic axon terminals in prelimbic cortex make contacts onto pyramidal neurons and, in particular, onto corticostriatal neurons as well as layer I inhibitory interneurons. Using stereology, we made quantitative estimates of contacts in uppermost prelimbic layer I onto dendrites of pyramidal neurons, corticostriatal neurons and layer I inhibitory interneurons. Prefrontal cortex has long been associated with decision making. Specifically, corticostriatal neurons in rat prelimbic cortex play an important role in cost–benefit decision making. Although recent experiments have detailed the physiology of this area in thalamocortical circuits, the extent of the impact of ventromedial thalamic input on corticostriatal neurons or layer I inhibitory interneurons has not been explored. Our quantitative anatomical results provide evidence that most ventromedial thalamic input to pyramidal neurons is provided to corticostriatal neurons and that overall more contacts are made onto the population of excitatory than onto the population of inhibitory neurons.

## Introduction

Ventromedial (VM) thalamic projection neurons extensively innervate cortical layer I (Herkenham 1979; Arbuthnott et al. 1990). Biane et al. (2016) optically stimulated axon terminals from the ventral motor thalamic nuclei, i.e., the ventromedial, ventrolateral and ventral anterior thalamic nuclei (Sieveritz et al. 2019), and recorded monosynaptic responses ex-vivo in rat primary motor cortex. After skilled grasp training, response amplitude of layer V neurons that control grasp related muscles in the distal forelimb was significantly larger compared to layer V neurons that control muscles in the proximal forelimb (Biane et al. 2016). Given that these neurons are targets of ventral motor thalamic projection neurons their axons may be involved in memory formation, a prerequisite for many behavioral tasks. In mice, VM projection neurons to a cortical region named anterior lateral motor cortex (ALM; from bregma AP + 2.5 mm, ML + 1.5 mm) make contacts onto apical dendritic tufts of layer VB pyramidal tract neurons that, in turn, project back to VM forming a thalamo-cortical-thalamic loop (Guo et al. 2018). Unilateral photoinhibition of ALM or VM activity during a delay phase, prior to making a response, impairs the performance of animals on a sensory discrimination task, highlighting the importance of this thalamocortical loop in planning a response (Guo et al. 2017). We previously suggested that VM projection neurons to other frontal cortical areas may be involved in regulating behaviors associated with those frontal cortical areas (Sieveritz et al. 2019). For example, optogenetic manipulation of prelimbic corticostriatal neurons in cost–benefit decision-making alters the preference of rats for a high cost-high reward option as compared to a low cost-low reward option, even though perception of cost and reward values remains unchanged (Friedman et al. 2015, 2017). Optical stimulation of VM axon terminals in prelimbic cortex evokes strong responses in layers II/III and layer V corticocortical neurons and weaker responses in layer V corticothalamic (CT) neurons (Collins et al. 2018). Corticocortical neurons, also known as intratelencephalic or corticostriatal neurons (CS), send collaterals to striatum (Wilson 1986, 1987; Gerfen et al. 2018). Considering that VM input evokes responses in these prelimbic CS neurons that are involved in cost–benefit decision-making, VM input may itself be involved in cost–benefit decision-making. Therefore, we developed a special interest in the innervation pattern of VM projection neurons to prelimbic cortex.

VM projection neurons to prelimbic cortex may also make contacts onto layer I inhibitory interneurons that recently received much interest in other cortical regions (Ma et al. 2014; Abs et al. 2018; Roth et al. 2016; Lam and Sherman 2019; Schuman et al. 2019; Senzai et al. 2019) and even in humans and primates (Gabbott 2016; Poorthuis et al. 2018). Cruikshank et al. (2012) reported that optogenetic stimulation of midline thalamic axon terminals in secondary motor cortex and anterior cingulate cortex evoked a response in layer I inhibitory interneurons in mice, but evidence for prelimbic cortex is sparse. Layer I inhibitory interneurons can, in turn, induce feedforward inhibition in cortical pyramidal neurons (Jiang et al. 2013, 2015). Thus, if VM axon terminals in prelimbic cortex make contacts onto pyramidal neurons as well as layer I inhibitory interneurons, VM input can potentially induce excitatory and/or inhibitory responses in prelimbic CS neurons. Such a circuit could be involved in subtle control of cortical output informing decision-making.

Here, we report anatomical evidence that thalamocortical projection neurons from VM to prelimbic cortex indeed make contacts onto dendrites of layer I inhibitory interneurons and pyramidal neurons, and specifically onto the dendritic tufts of CS neurons. Furthermore, we show that VM projection neurons preferentially target CS neurons and provide a quantitative comparison of the frequency of contacts onto dendrites of these three kinds of cortical cell types. In addition, we used immunohistochemistry to confirm that ventral motor thalamic axon terminals are glutamatergic supported by both vesicular glutamate transporter 1 (VGluT1) and 2 (VGluT2).

## Methods

### Ethical approval

All applicable international, national, and/or institutional guidelines for the care and use of animals were followed. All procedures performed in studies involving animals were in accordance with the ethical standards of the Okinawa Institute of Science and Technology Graduate University and were approved by the Animal Care and Use Committee of the Okinawa Institute of Science and Technology Graduate University (protocols #2016–131 and #2018–212).

### Experimental design

**Fig. 1:**
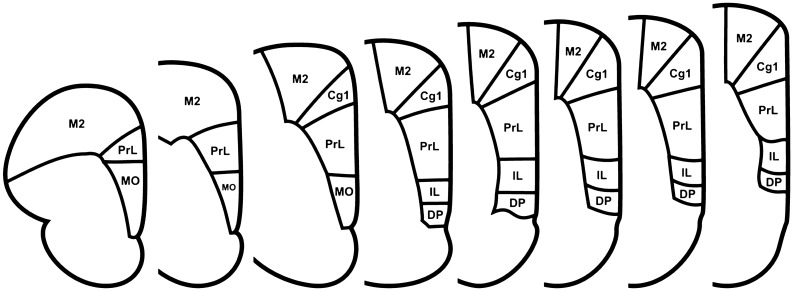
The extent of prelimbic cortex was defined in accordance with borders presented in a rat brain atlas (Paxinos and Watson 2004). The boundaries of prelimbic cortex based on this definition are illustrated in this figure (*PrL* prelimbic cortex, *M2* secondary motor cortex, *MO* medial orbital cortex, *Cg1* cingulate cortex, area 1, *IL* infralimbic cortex, *DP* dorsal peduncular cortex)

In twelve 9- to 10-week-old male Sprague–Dawley rats, we deposited small amounts of anterograde tracer in VM or the ventral motor thalamic nuclei and studied the axon terminals in uppermost layer I of prelimbic cortex. The extent of VM, the ventral motor thalamic nuclei and prelimbic cortex was defined in accordance with borders presented in a rat brain atlas (Paxinos and Watson 2004). The extent of prelimbic cortex is illustrated in Fig. 1. The location of VM and the ventral motor thalamic nuclei was determined based on histological markers. The location of prelimbic cortex was determined based on anatomical markers and distance from pia mater. Immunohistochemical methods were used to probe for proteins that mark glutamatergic and GABAergic synapses in ascending ventral motor thalamic projection neurons in prelimbic cortical layer I, i.e., VGluT1 VGluT2 and glutamate decarboxylase 67 (GAD67), as well as to look for superimposition between VM axon terminals and expected postsynaptic structures in ipsilateral prelimbic cortical layer I. Superimpositions, or contacts, were defined as an overlap between axon terminals and these proteins or postsynaptic structures of at least four voxels in *x*- and *y*- direction in single optical planes of microscope images.

To visualize expected postsynaptic structures, we performed immunohistochemistry for (1) microtubule-associated protein 2 (MAP2), a protein expressed in somata and dendrites of cortical pyramidal neurons (Bernhardt and Matus 1984), (2) regulator of G-protein signaling 14 (RGS14), a protein that marks somata and postsynaptic sites along dendrites of intratelencephalic CS neurons in prelimbic cortex (Gerfen et al. 2013), and (3) five-hydroxytryptamine 3a receptor (5HT3aR), a protein expressed by 99% of cortical layer I inhibitory interneurons (Rudy et al. 2011; Feldmeyer et al. 2018). Stereological counting of above-mentioned superimpositions allowed us to compare the likelihood of each kind of contact onto dendrites of pyramidal neurons, CS neurons or 5HT3aR-positive inhibitory interneurons in layer I.

Antibodies for VGluT1, VGluT2 and GAD67 do not mark entire axons, while antibodies for RGS14 and 5HT3aR do not mark entire dendrites. However, GAD67 (Kanaani et al. 2010, 2015), VGluT1 and VGluT2 are expressed in presynaptic elements (Fremeau et al. 2001). Thus, antibodies for these proteins will label expression at presynaptic sites and are suitable to explore expression in VM axon terminals. Evidence suggests that the receptor-associated proteins RGS14 (Squires et al. 2018) and 5HT3aR are primarily expressed at synaptic sites (Huang et al. 2004). More specifically, electrophysiological evidence suggests that at least 5HT3aR is primarily expressed in postsynaptic compartments in cerebral cortex (Zhou and Hablitz 1999; Ferezou et al. 2002). Because contacts between incoming VM axon terminals and dendrites of prelimbic cortical neurons will occur between pre- and postsynaptic sites, markers expressed only at synaptic sites and, specifically, at postsynaptic sites rather than in entire dendrites are sufficient to identify these contacts. The unique spatial arrangement of these markers in the cerebral microspace strongly suggests that we are highlighting structures involved in synaptic contacts between incoming VM axon terminals onto dendrites of postsynaptic neurons.

### Verification of the used antibodies

Although specificity of the RGS14 and 5HT3aR antibodies that were used in this study has been confirmed by the supplier using immunoblot and immunohistochemistry, use of these antibodies to visualize somata and synaptic sites along dendrites of prelimbic CS neurons or 5HT3aR-positive inhibitory interneurons in prelimbic cortical layer I, has not been reported previously. As we were using these antibodies to visualize postsynaptic structures, we first tested whether these antibodies are indeed expressed pre- or postsynaptic in prelimbic cortical layer I. We performed immunohistochemistry in 80 µm-thick coronal sections from prefrontal cortex of three 12-week-old male Sprague–Dawley rats for RGS14, synaptophysin one (SOP) and postsynaptic density protein 95 (PSD95) or for 5HT3aR, SOP and PSD95. We verified for each animal in two image stacks taken in prelimbic cortex (for details see “Microscopy”) that expression of both markers, RGS14 and 5HT3aR, was restricted to postsynaptic sites.

We next confirmed that the used antibodies were suitable to visualize somata and synaptic sites along dendrites of prelimbic CS neurons or 5HT3aR-positive inhibitory interneurons in prelimbic cortical layer I. RGS14 is expressed in the nuclei of a variety of cells in cortex (Lopez-Aranda et al. 2006), but somatic and dendritic expression of RGS14 in prelimbic cortex was reported to be limited to CS neurons (Gerfen et al. 2013). CS neurons are a specific type of pyramidal neuron. Hence, we first verified that in prelimbic cortex RGS14 is indeed expressed in dendrites of pyramidal neurons. We performed immunohistochemistry for MAP2, a marker for dendrites of pyramidal neurons, and RGS14 in 80 µm-thick coronal sections taken from prefrontal cortex of the same three 12-week-old male Sprague–Dawley rats. We confirmed for each animal in two image stacks taken in prelimbic layers II/III (for details see “Microscopy”) that RGS14 was expressed along dendrites of pyramidal neurons marked with MAP2.

To verify that somatic and dendritic expression of RGS14 in prelimbic cortex is limited to CS neurons, we retrogradely labeled CS neurons in seven and CT neurons in four 9- to 10-week-old male Sprague–Dawley rats using choleratoxin subunit B. After a 14-day survival period we took 80 μm-thick coronal sections from prefrontal cortex and performed immunohistochemistry for RGS14 and choleratoxin subunit B. For each animal, two image stacks of retrogradely labeled CS and CT neurons were taken in prelimbic cortex (for details see “Microscopy”). To control for false-negative labeling, we verified that retrogradely labeled CS neurons in rat prelimbic cortex are RGS14-positive. To control for false-positive labeling we verified that retrogradely labeled CT neurons in rat prelimbic cortex are RGS14-negative.

In rats, 90% of 5HT3aR-positive neurons in cortex and hippocampus are GABAergic (Morales et al. 1996). In cortex 5HT3aR reportedly is only expressed in inhibitory interneurons (Rudy et al. 2011). In mice, 99% of inhibitory interneurons in cortical layer I express 5HT3aR (Rudy et al. 2011; Feldmeyer et al. 2018), and all interneurons in cortical layer I are GABAergic and express GAD67 (Retaux et al. 1993). Therefore, all 5HT3aR-positive neurons in cortical layer I express GAD67, while the vast majority of GAD67-positive neurons in cortical layer I (99%) are expected to express 5HT3aR.

We performed immunohistochemistry for GAD67 and 5HT3aR in 80 µm-thick coronal sections taken from prefrontal cortex of six male 12-week-old Sprague–Dawley rats and for each animal took one image stack in prelimbic layer I as well as in secondary motor cortex layer I (for details see “Microscopy”; results for secondary motor cortex are not shown but are available on figshare at https://dx.doi.org/10.6084/m9.figshare.7039376). To confirm that no false-negative staining occurs, we verified that all GAD67-positive neuronal cell bodies in prelimbic and secondary motor cortex layer I expressed 5HT3aR. To further confirm that no false-positive staining occurs, we verified that all GAD67-negative neuronal cell bodies in prelimbic and secondary motor cortex layer I did not express 5HT3aR.

### Retrograde and anterograde tracing

Male Sprague–Dawley rats, 9–10 weeks of age, were anesthetized with 5% isoflurane delivered with room air (0.5–1 L/min; Small Animal Anesthetizer, Muromachi, MK-A100, Japan). Animals were positioned in a stereotaxic frame (Narishige, SR-5R-HT, Japan) and anesthesia was maintained at 2% isoflurane delivered with room air (0.5–1 L/min). In retrograde tracing experiments, animals received bilateral injections of 300 nL cholera toxin subunit B (1 mg/mL, Sigma Aldrich, #C9903) into the dorsomedial striatum (from interaural zero AP + 9.89 to + 13.15, ML + 2.4/− 2.4, from bregma DV − 4.0 mm) or a unilateral injection of 300 nL cholera toxin subunit B (1 mg/mL, Sigma Aldrich, #C9903) into ventral motor and adjacent thalamic nuclei (from interaural zero AP + 7.14 to + 9.55, ML − 1.2, from dura DV − 6.56 or − 6.6 mm). In anterograde tracing experiments, animals received unilateral injections of 50–70 nL AAV5-CAG-ArchT-GFP (titer ≥ 7 × 10^12^ vg/mL; gift from Edward Boyden and purchased through UNC Vector Core; now commercially available from Addgene; viral preparation #29777-AAV5; https://n2t.net/addgene:29777; RRID:Addgene_29777) or 50–70 nL AAV5-CAG-GFP (titer ≥ 7 × 10^12^ vg/mL; gift from Edward Boyden and purchased through UNC Vector Core; now commercially available from Addgene; viral preparation #37825-AAV5; https://n2t.net/addgene:37825; RRID:Addgene_37825) into ventral motor thalamic nuclei, or of 10–20 nL biotinylated dextran conjugated with tetramethylrhodamine (mini-ruby; 10,000 molecular weight, 50 mg/mL, Thermo Fisher Scientific, #D3312) into VM. Injections were performed with a Hamilton syringe (Neuros Syringe, 0.5 μL, Neuros Model 7000.5 KH, point style three, Hamilton, #65457–01, United Kingdom) at an injection speed of 100 nL/min and the syringe remained at the target location for 10 min after the injection before it was slowly retracted from the brain. Animals injected with AAV5-CAG-ArchT-GFP or AAV5-CAG-GFP were perfused after a survival period of 25–26 days. All other animals were perfused after a 14-day survival period.

### Perfusion

Rats were anesthetized with approximately 10 ml isoflurane delivered on tissue paper and medetomidine hydrochloride (1.0 mg/kg, intraperitoneal), before being transcardially perfused with either 100 mL 0.1 M phosphate buffer with 2 mg/100 mL heparin (4 °C, pH7.4) or 100 mL of 10% sucrose diluted in Milli-Q, both followed by 150 mL Lana’s fixative (room temperature, 4% depolymerized paraformaldehyde and 14% saturated picric acid solution, Sigma Aldrich, #P6744-1GA, in 0.16 M phosphate buffer, pH7.4). Brain tissue was post-fixed for at least 36 h and afterward stored in a 50/50 mixture of Lana’s fixative and 20% sucrose in phosphate-buffered saline (PBS) until sectioning, i.e., a minimum of 36 h and up to 3 weeks.

### Immunohistochemistry

80 μm-thick coronal sections were taken from prefrontal cortex (interaural zero + 11.52 to + 13.68 mm; in accordance with borders presented in a rat brain atlas, Paxinos and Watson 2004), dorsomedial striatum (interaural zero + 8.40 to + 11.28 mm; in accordance with borders presented in a rat brain atlas, Paxinos and Watson 2004) and ventral motor thalamic nuclei (interaural zero + 4.80 to + 8.76 mm; in accordance with borders presented in a rat brain atlas, Paxinos and Watson 2004) using a freezing microtome (Yamato, REM-710, Japan). Sections were washed in PBS for 3 × 5 min before being incubated in 20% goat serum (Vector Laboratories, #S-1000) in PBS, containing 0.05% sodium azide and 0.15–0.3% Triton X-100 (Sigma Aldrich, #234729-100ML), at room temperature for 1 h. Afterward, sections were incubated for 18–36 h at 4 °C in primary antibody solution containing primary antibodies diluted in PBS, 0.05% sodium azide and 0.15–0.3% Triton X-100. Next, sections were washed in PBS for 3 × 5 min before being incubated in secondary antibody solution containing secondary antibodies diluted in PBS, 0.05% sodium azide and 0.3% Triton X-100, at room temperature for 2–3 h. Finally, sections were washed in PBS for 4 × 5 min. Dilutions of primary and secondary antibodies, the concentration of Triton X-100 in primary antibody solutions, and the incubation time of sections varied depending on the antibodies used and are provided in Table 1. Sections were mounted on glass slides in several drops of mounting medium and sealed with a coverslip. The used mounting media were UltraCruz Hard-set Mounting Medium with DAPI (Santa Cruz, #sc-359850) or Vectashield HardSet Mounting Medium with DAPI (Vector Laboratories, #H-1500) for DNA counterstaining of cell nuclei, or if Alexa Fluor 405 was used as a secondary antibody VectaMount AQ (Vector Laboratories, #H-5501). Secondary antibodies were acquired from Millipore or Invitrogen with exception of Alexa Fluor 405 goat anti-rabbit, which was acquired from abcam.

**Table 1:** Primary and secondary antibodies used in immunohistochemistry

Primary antibody	Secondary antibody
Rabbit anti-choleratoxin subunit-B (abcam, #ab34992, RRID: AB_726859): 1:1000 dilution, 0.15–0.3% Triton X-100, 18-36 h incubation time	Alexa Fluor 594 goat anti-rabbit: 1:200 dilution, 2–3 h incubation time
Mouse anti-GAD67 (Millipore, #MAB5406, RRID: AB_2278725): 1:1000 dilution, 0.15–0.3% Triton X-100, 18-36 h incubation time	Alexa Fluor 488/594/633 goat anti-mouse: 1:200 dilution, 2–3 h incubation time
Guinea pig anti-VGluT1 (Millipore, #AB5905, RRID: AB_2301751): 1:500 dilution, 0.3% Triton X-100, 18 h incubation time	Alexa Fluor 594 goat anti-guinea pig: 1:200 dilution, 3 h incubation time
Rabbit anti-VGluT2 (Sigma Aldrich, #V2514, RRID: AB_477611): 1:1000 dilution, 0.3% Triton X-100, 18 h incubation time	Alexa Fluor 405 goat anti-rabbit: 1:200 dilution, 3 h incubation time
Rabbit anti-PSD95 (abcam, #ab76115, RRID: AB_1310620): 1:200 dilution, 0.15% Triton X-100, 36 h incubation time	Alexa Fluor 405 goat anti-rabbit: 1:200 dilution, 2 h incubation time
Mouse anti-PSD95 (Invitrogen, #MA1-046, RRID: AB_2092361): 1:1000 dilution, 0.15% Triton X-100, 36 h incubation time	Alexa Fluor 405 goat anti-mouse: 1:200 dilution, 2 h incubation time
Guinea pig anti-synaptophysin 1 (Synaptic systems, #101 004, RRID: AB_1210382): 1:500 dilution, 0.15% Triton X-100, 36 h incubation time	Alexa Fluor 594 goat anti-guinea pig: 1:200 dilution, 2 h incubation time
Rabbit anti-MAP2 (abcam, #ab32454, RRID: AB_776174): 1:500 dilution, 0.15% Triton X-100, 36 h incubation time	Alexa Fluor 488 goat anti-rabbit: 1:200 dilution, 2 h incubation time
Mouse anti-RGS14 (NeuroMabs, #73–170, RRID: AB_10698026): 1:500 dilution, 0.15% Triton X-100, 36 h incubation time	Alexa Fluor 488 goat anti-mouse: 1:200 dilution, 2 h incubation time
Rabbit anti-5HT3aR (Invitrogen, #PA1-41,033, RRID: AB_2248968): 1:500 dilution, 0.15% Triton X-100, 36 h incubation time	Alexa Fluor 488 goat anti-rabbit: 1:200 dilution, 2 h incubation time
Chicken anti-MAP2 (abcam, #ab5392, RRID: AB_2138153): 1:500 dilution, 0.15% Triton X-100, 36 h incubation time	Alexa Fluor 633 goat anti-chicken: 1:200 dilution, 2 h incubation time

A summary of dilutions of primary and secondary antibodies in respective antibody solutions, the concentration of Triton X-100 in primary antibody solutions, and incubation times of sections

### Microscopy

Laser confocal scanning microscopy was performed with a Zeiss LSM 780 microscope at room temperature. Images were taken with a 10 × objective with a numerical aperture (NA) of 0.45; a 20 × objective with a NA of 0.8; a 63 × oil objective with a NA of 1.46 or a 100 × oil objective with a NA of 1.46 (all objectives from Zeiss). The 63 × and the 100 × objective were used with fluorescence-free Immersol immersion oil 518F from Zeiss. An overview of the excitation and emission peak of used fluorochromes, the light source and filter set used for excitation, and the emission range used are provided in Table 2.

**Table 2:** Used fluorochromes

Fluorochrome	Excitation	Emission	Light source	Filter set	Emission range
DAPI	358 nm	461 nm	405–30 laser diode	MBS 488/561/633 dichroic beam splitter; MBS − 405 dichroic beam splitter	Simultaneous use of Alexa Fluor 488: 410–495 nm. Otherwise: 410–579 nm
Alexa Fluor 405	401 nm	421 nm	405–30 laser diode	MBS 488/561/633 dichroic beam splitter; MBS − 405 dichroic beam splitter	410–489 nm
Alexa Fluor 488	496 nm	519 nm	Argon laser	MBS 488 dichroic beam splitter	Simultaneous use of Alexa Fluor 594: 489–588 nm. Otherwise: 493–630 nm
GFP	395 nm	509 nm	Argon laser	MBS 488 dichroic beam splitter	489–588 nm
Tetramethyl-Rhodamine	557 nm	576 nm	DPSS 561–10 laser	MBS 458/561 dichroic beam splitter	568–691 nm
Alexa Fluor 594	590 nm	617 nm	DPSS 561–10 laser	MBS 458/561 dichroic beam splitter	Simultaneous use of Alexa Fluor 633: 587–633 nm. Otherwise: 585–735 nm
Alexa Fluor 633	633 nm	647 nm	HeNe633 laser	MBS 488/561/633 dichroic beam splitter	638–747 nm

Overview of the excitation and emission peak of used fluorochromes, the light source and filter set used for excitation, and the emission range used

All tiled overview images were acquired with 10% overlap between tiles. Image stacks acquired for systematic random sampling consisted of either 1 × 18 or 2 × 18 tiles. Image stacks covered the entire uppermost 40 µm of prelimbic cortex in each slice imaged. Boundaries of prelimbic cortex were determined in accordance with those presented in a rat brain atlas (Paxinos and Watson 2004) and location of prelimbic cortex in each section was determined based on histological markers and distance from pia mater. Each image stack spanned five single focal plane images with 2.32 µm between single focal plane images. Image stacks with 1 × 18 tiles were a total of 134.95 × 2199.70 × 11.60 μm and image stacks with 2 × 18 tiles were a total of 256.41 × 2199.70 × 11.60 μm. The size of each voxel was 0.1318 × 0.1318 × 2.32 μm. However, even though voxels were 2.32 µm long, according to an equation to determine the full-width-of-half-maximum for an Airy disk provided by Zeiss (Toomre et al. 2019) the actual span of each single focal plane image was approximately 420–460 nm. Superimpositions between ventral motor thalamic axon terminals in the uppermost 40 µm of prelimbic cortex and postsynaptic structures were quantified in each of the five single focal plane images in the image stack using a technique known as systematic random sampling (for details see “Systematic Random Sampling”). Single focal plane images were not combined into a maximum intensity projection.

Expression of VGluT1 and VGluT2, but absence of GAD67 in VM axon terminals in prelimbic cortex was confirmed in image stacks that consisted of 10 or 11 single focal plane images with 0.58 µm between single focal plane images. The number of single focal plane images as well as distance between single focal plane images were adapted from (Lei et al. 2013), who were examining presence and absence of neurotransmitters in thalamostriatal axon terminals. Image stacks with 10 single focal plane images had a total size of 134.9511 × 134.9511 × 5.22 μm and those with 11 single focal plane images had a total size of 134.9511 × 134.9511 × 5.80 μm. The size of each voxel was 0.1318 × 0.1318 × 0.58 μm. However and as pointed out above even though voxels were 0.58 µm long, according to an equation to determine the full-width-of-half-maximum for an Airy disk provided by Zeiss (Toomre et al. 2019) the actual span of each single focal plane image was approximately 420–460 nm. Images cropped from maximum intensity projections performed across each image stack (methods adapted from (Lei et al. 2013) as well as, to examine absence of neurotransmitters, images cropped from single focal plane images spanning 0.58 μm are shown.

To determine whether RGS14 and 5HT3aR are expressed pre- or postsynaptic, we took two image stacks for each animal consisting of 10 single focal plane images with 0.14 μm between single focal plane images in prelimbic cortical layer I. The total size of each image stack was 134.9511 × 134.9511 × 1.40 μm and the size of each voxel was 0.066 × 0.066 × 0.14 μm. To determine whether RGS14 is expressed in dendrites of prelimbic pyramidal neurons that were marked with MAP2, for each animal one image stacks consisting of 10 single focal plane images with 0.13 μm between single focal plane images was taken in prelimbic cortical layers II/III. The total size of each image stack was 134.9511 × 134.9511 × 1.30 μm and the size of each voxel was 0.066 × 0.066 × 0.13 μm. All other image stacks span 20 single focal plane images with 0.29 μm between single focal plane images. The total size of each of these image stacks was 134.9511 × 134.9511 × 5.86 μm and the size of each voxel was 0.1318 × 0.1318 × 0.2893 μm. To determine if VM axon terminals in the uppermost 40 μm of prelimbic cortex superimpose with dendrites of pyramidal neurons, CS neurons or 5HT3aR-positive inhibitory interneurons in cortical layer I we looked for superimpositions in these single focal plane images that each were 0.29 µm thick. Single focal plane images were not combined into a maximum intensity projection. Images shown below were cropped from all the above mentioned single focal plane images spanning 0.29 μm, 0.13 μm or 0.14 μm.

Images were acquired using the ZEN 2011 SP7 FP1 software (Zeiss, black edition, version 14.0.8.201). Brightness and contrast were adjusted using ZEN 2011 SP7 FP1 software (Zeiss, black edition, version 14.0.8.201) and the “Enhance Contrast” option in Fiji (ImageJ version 1.51n or 1.52p). When necessary, brightness and contrast were adjusted separately for each channel using the mentioned software. 3D-reconstructions from these brightness- and contrast-adjusted images were performed using Imaris (version 9.3.0 64x). For final publication, brightness and contrast of images were additionally adjusted using Adobe Photoshop CS5 Extended (version 12.0.4 × 64). Adjustments to brightness and contrast were always applied equally across the entire image.

In addition, histological verification of AAV5-CAG-ArchT-GFP and AAV5-CAG-GFP injections into ventral motor thalamic nuclei was performed with a fixed stage BX51WI upright microscope from Olympus. A 100 W mercury lamp was used as light source. Alexa Fluor 594 was visualized using an excitation filter with a peak at 555 nm and a bandwidth of 25 nm, and an emission filter with a peak at 605 nm and a bandwidth of 25 nm. GFP was visualized using an excitation filter with a peak at 484 nm and a bandwidth of 15 nm, and an emission filter with a peak at 517 nm and a bandwidth of 30 nm. Images were acquired with a 4 × objective with a NA of 0.16 using the Neurolucida software (MBF Bioscience).

### Systematic random sampling

**Fig. 2:**
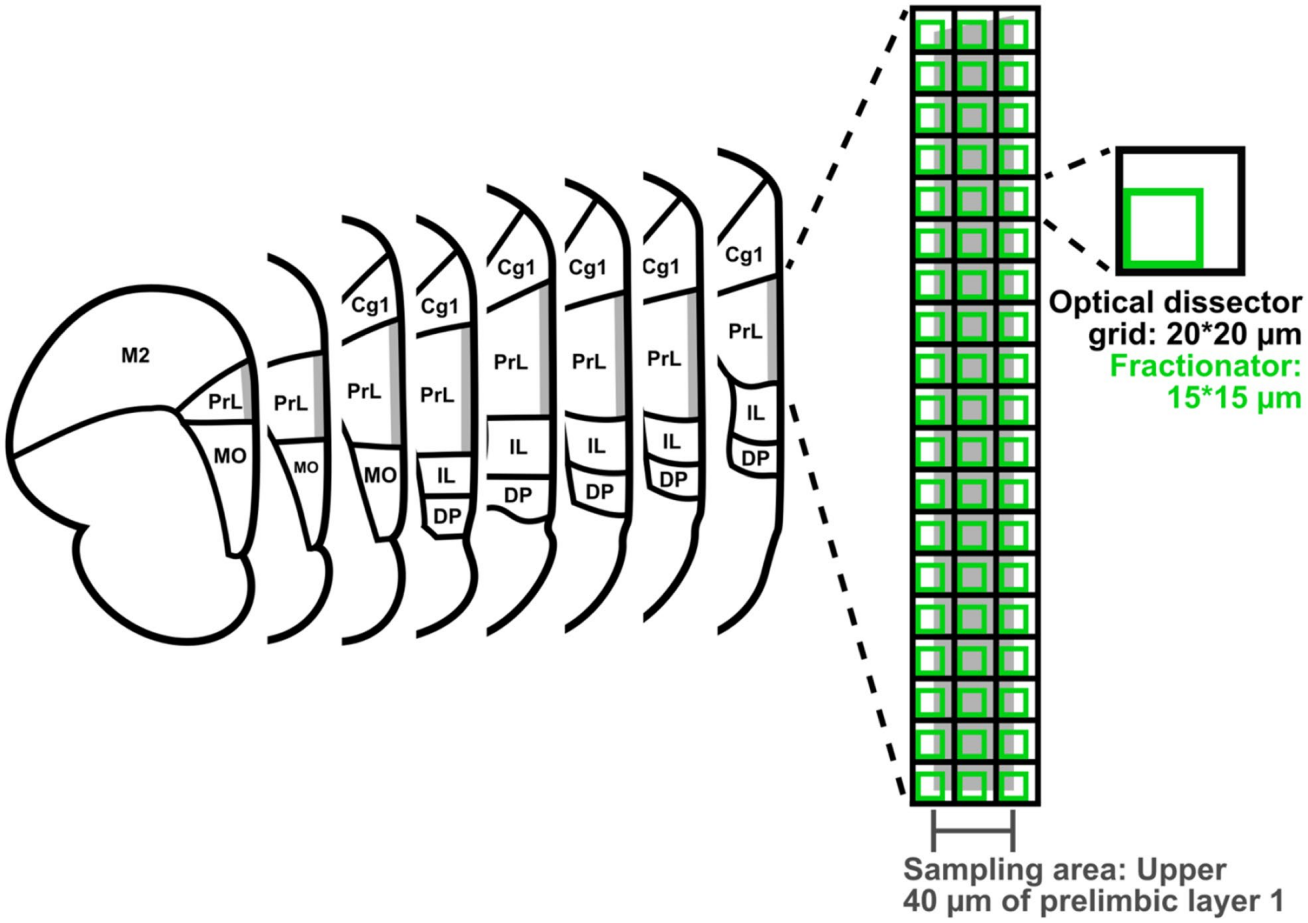
Stereology. The sampling area, defined a priori, consisting of the uppermost 40 µm of prelimbic cortex in each third section is shaded in grey. In each image stack, the sampling area was outlined in the optical fractionator workflow in Stereo Investigator (MBF Biosciences) and the workflow placed the optical dissector grid randomly across it. The fractionator was placed systematically along the optical dissector grid to select sampling sites and define the size of each sampling site

The total number of superimpositions between VM axon terminals in the uppermost 40 μm of prelimbic cortical layer I and dendrites of pyramidal neurons, CS neurons or 5HT3aR-positive inhibitory interneurons was estimated using a technique known as systematic random sampling (Keuker et al. 2001; Schmitz and Hof 2005; Altunkaynak et al. 2012; Golub et al. 2015). Given that in sections taken at 80-µm thickness, prelimbic cortex spans only about 24 sections and we had systematically chosen a priori to sample from every third section, the first section for sampling was not chosen randomly. Instead every third section that contained prelimbic cortex and had been stained for each respective marker was included for sampling. The region of interest was also chosen systematically a priori and defined as the uppermost 40 μm of prelimbic cortex, because previous evidence indicates that VM projections primarily target uppermost prelimbic layer I (Arbuthnott et al. 1990). The extension of prelimbic cortex was defined in accordance with borders presented in a rat brain atlas (Paxinos and Watson 2004) and as shown in Figs. 1 and 2. The location of prelimbic cortex in each section was determined based on histological markers and distance from pia mater. Systematic random sampling was performed using image stacks acquired in every third section that spanned five single focal plane images with 2.32 µm between single focal plane images and a total of 11.6 μm (for details see “Microscopy”). Superimpositions between incoming VM axon terminals and postsynaptic structures were sampled using an optical fractionator. The optical fractionator combines an optical dissector, a systematic uniform sampling scheme to select sampling sites, with a fractionator, a three-dimensional probe that systematically defines the boundaries of each area to be sampled (Keuker et al. 2001; Schmitz and Hof 2005; Golub et al. 2015). While both the fractionator and optical dissector were chosen systematically, the placement of the optical dissector grid across the area of interest was chosen randomly.

The total number of co-localizations in the tissue was determined based on the following equation (Keuker et al. 2001):

1 $$N = \sum {Q^{ - } \times \frac{1}{{{\text{ssf}}}} \times \frac{1}{{{\text{asf}}}} \times \frac{1}{{{\text{tsf}}}}} ,$$

where ∑*Q*^−^ corresponds to the total number of superimpositions counted, the sampling section fraction (ssf) corresponds to the proportion of sections that are sampled, i.e., every third section, the area sampling fraction (asf) corresponds to the proportion of the area sampled in sections compared to the total area taken up by the region of interest, i.e., the area covered by the fractionator placed along the optical dissector to determine sampling areas in proportion to the upper 40 µm of prelimbic cortex, and the thickness sampling fraction (tsf) corresponds to the proportion in height sampled in each section, i.e., 8 µm, compared to the average thickness of the mounted sections. The average thickness of each mounted section was measured when sections were imaged and manually entered when running the optical fractionator workflow.

Systematic random sampling was performed using the optical fractionator workflow in Stereo Investigator (MBF Bioscience). The area of interest was outlined electronically in the software for each image stack and the workflow placed the optical dissector grid randomly across it. We sampled image stacks taken in every third section using a fractionator of 15 × 15 × 8 μm, which was placed along an optical dissector grid of 20 × 20 μm (Keuker et al. 2001; Schmitz and Hof 2005; Golub et al. 2015). Selection of area of interest, random placement of the dissector grid across the area of interest and the placement of the systematically chosen fractionator along the optical dissector grid are illustrated in Fig. 2.

### Statistical analysis

Parameters for systematic random sampling were chosen so that the Gundersen coefficient of error (*m* = 1) was smaller than or equal to 0.1 (Gundersen and Jensen 1987; Gundersen et al. 2003). We calculated the mean and standard error of the mean of estimated total numbers of superimpositions across all seven animals. We further calculated the ratio of estimated contacts made by VM projection neurons in the uppermost 40 μm of prelimbic cortical layer I onto CS neurons as compared to pyramidal neurons and of contacts made onto 5HT3aR-positive inhibitory interneurons as compared to pyramidal neurons. To verify that significantly fewer contacts are made onto dendrites of CS neurons or layer I inhibitory interneurons as compared to contacts made onto dendrites of pyramidal neurons non-parametric Wilcoxon signed-rank tests were calculated in *R*. No further statistical analysis of the data was performed.

### Data availability

**Fig. 3:**
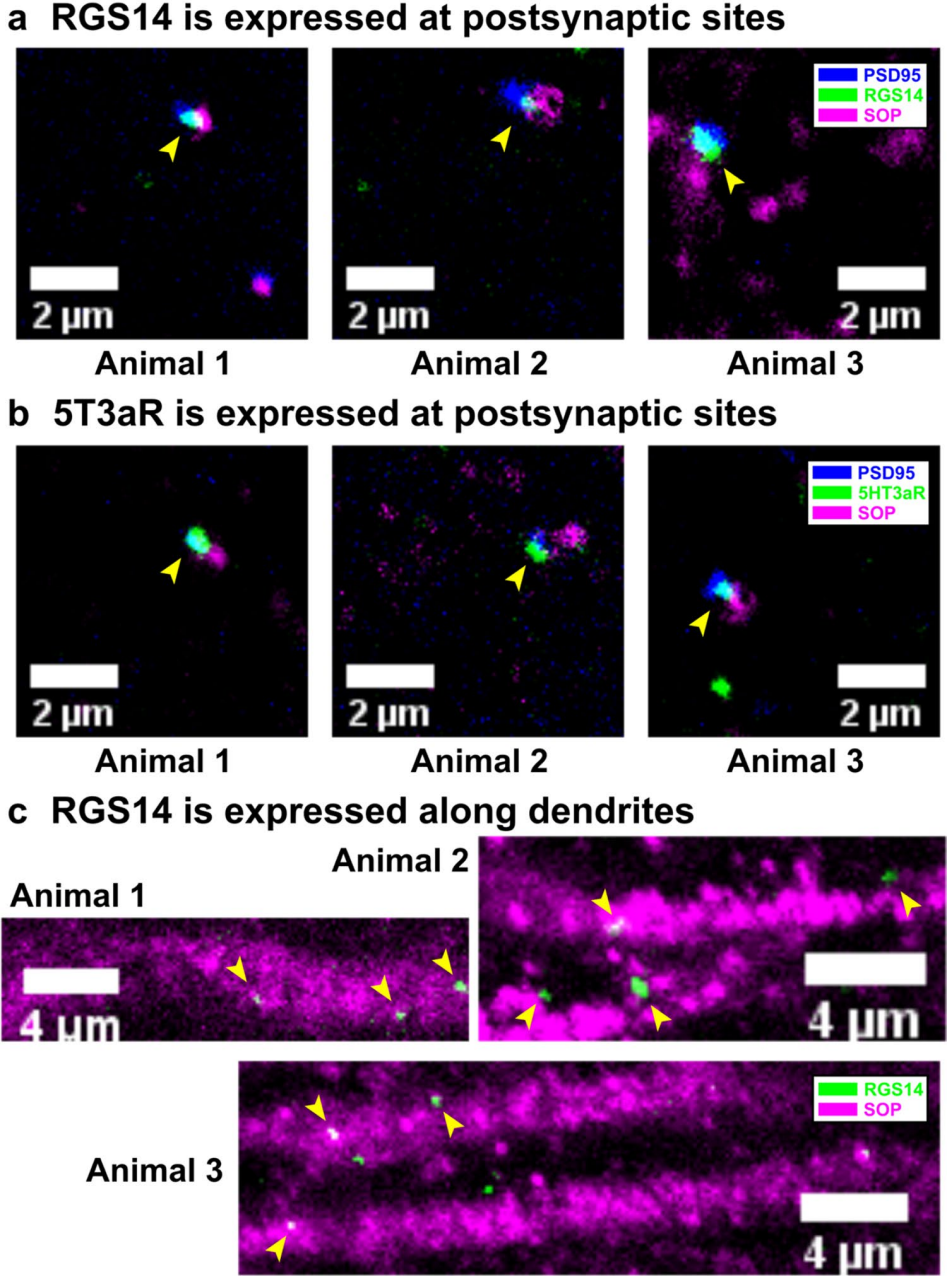
Expression of RGS14 and 5HT3aR at postsynaptic sites, and expression of RGS14 along dendrites of pyramidal neurons. **a** In prelimbic cortical layer I RGS14 (green) and **b** 5HT3aR (green) are expressed at postsynaptic sites marked with PSD95 (blue) rather than at presynaptic sites marked with SOP (magenta). Yellow arrowheads mark expression of RGS14 and 5HT3aR in postsynaptic sites. **c** Furthermore, in prelimbic cortical layers II/III RGS14 (green) is expressed along dendrites of pyramidal neurons marked with MAP2 (magenta). Yellow arrowheads mark expression of RGS14 along dendrites. Animal numbers do not correspond to animal numbers used in later figures

**Fig. 4:**
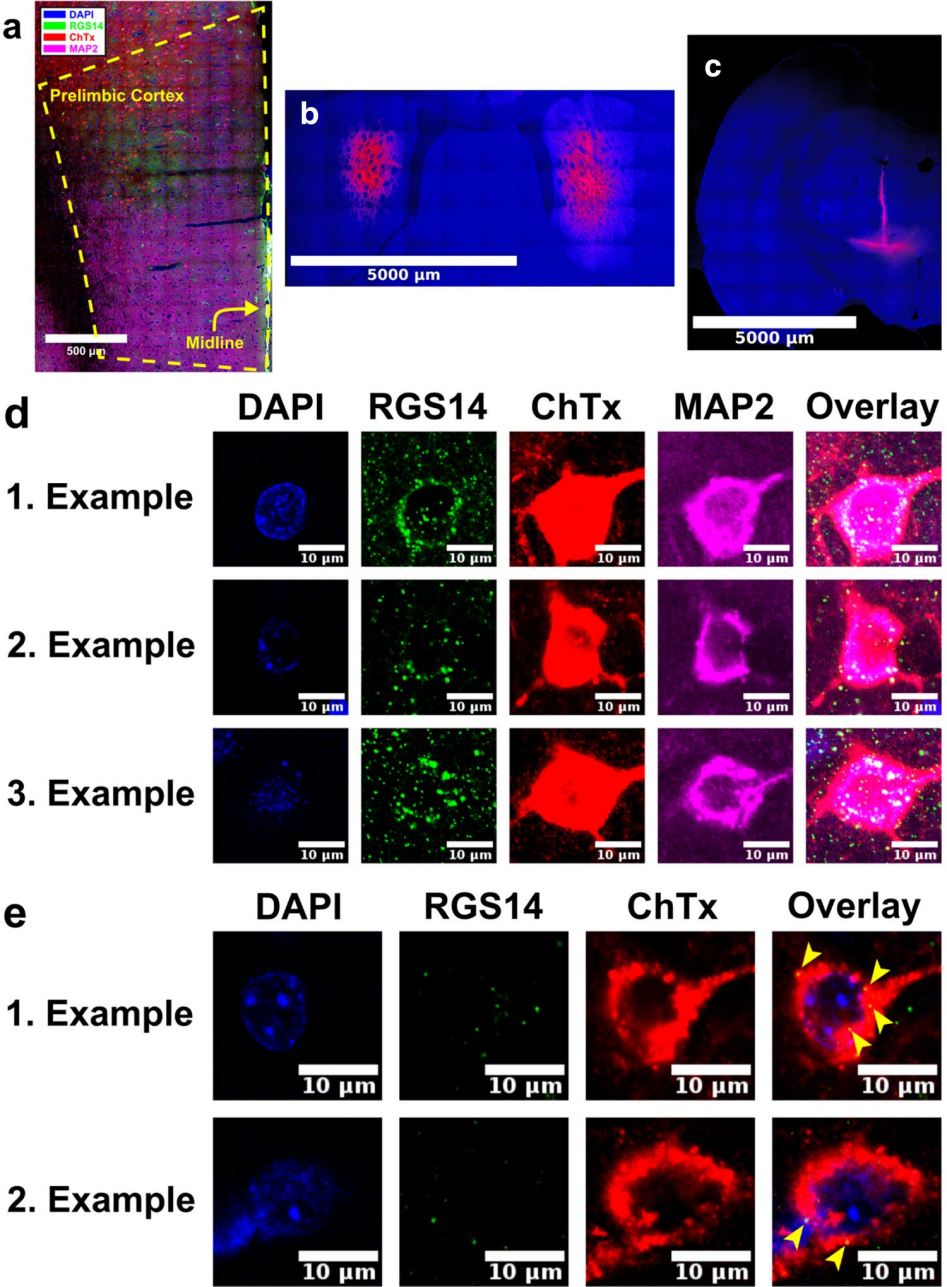
RGS14 expression in prelimbic cortex. **a** CS neurons that were retrogradely labeled with choleratoxin subunit B (ChTx, red) express RGS14 (green) and MAP2 (magenta) in prelimbic cortex, which is outlined by the yellow dashed line. **b** Injection site of choleratoxin subunit B (red) in dorsomedial striatum. **c** Injection site of choleratoxin subunit B (red) in ventral motor and adjacent thalamic nuclei. **d** CS neurons in prelimbic cortex that were retrogradely labeled with choleratoxin subunit B (ChTx; red) from dorsomedial striatum express RGS14 (green) and MAP2 (magenta), while **e** CT neurons do not express RGS14 (green) in the somata. Yellow arrowheads mark sparse expression of RGS14 along the outer membrane of the cell nuclei or in the somata that was observed in very few CT neurons in prelimbic cortex. Cell nuclei are counterstained with DAPI (blue)

**Fig. 5:**
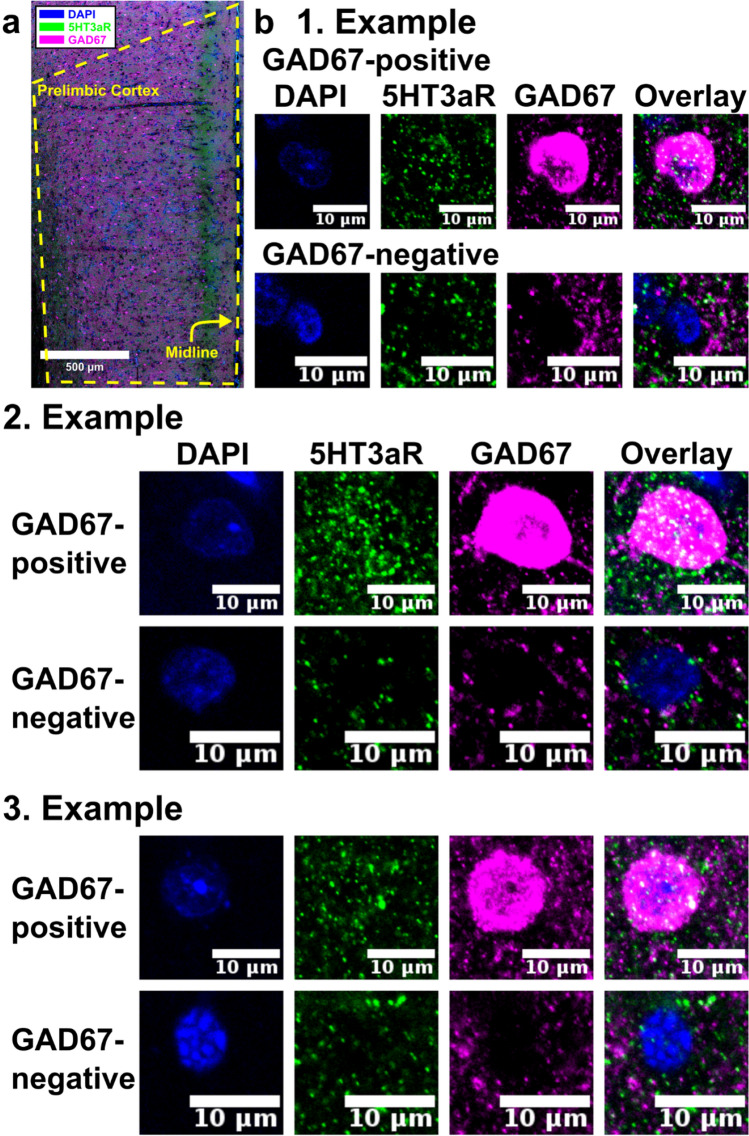
5HT3aR expression in prelimbic cortex. **a** Expression of 5HT3aR (green) and GAD67 (magenta) in prelimbic cortex that is outlined by the yellow dashed line. **b** In prelimbic layer I inhibitory interneurons that were labeled with GAD67 (GAD67-positive; magenta) express 5HT3aR (green), while those that do not express GAD67 (GAD67-negative; magenta) do not express 5HT3aR (green). Cell nuclei were counterstained with DAPI (blue)

**Fig. 6:**
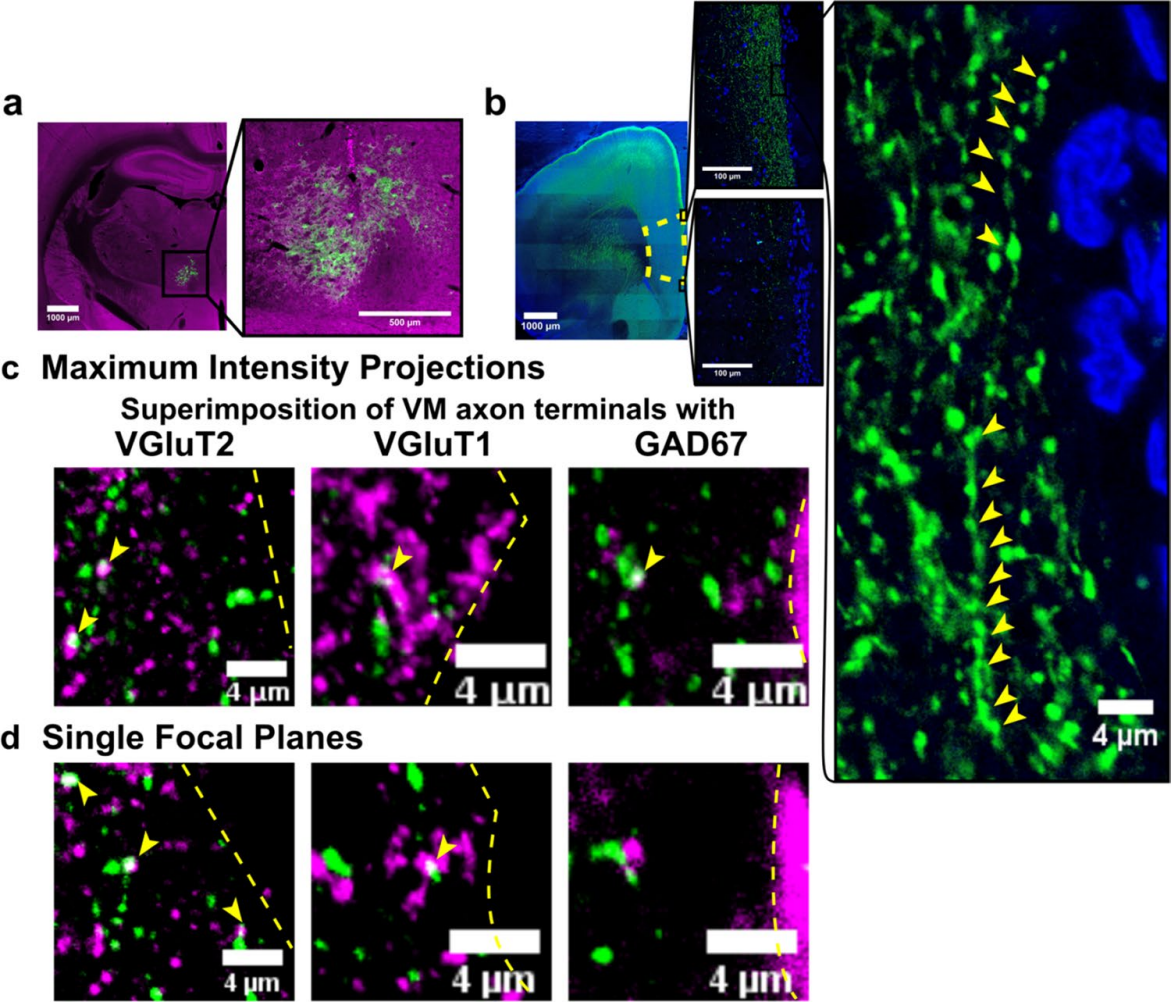
VM axon terminals in prelimbic cortex express VGluT2 and to less extent VGluT1 but not GAD67. **a** Injection of the anterograde tracer AAV5-CAG-ArchT-GFP (green) into ventral motor thalamus, which was labeled with GAD67 (magenta), **b** labeled ventral motor thalamic axon terminals (green) in a prefrontal cortical section (left). Prefrontal cortex in this section is outlined by a yellow dashed line. Specifically, ventral motor thalamic axon terminals (green) spanned the full length from the dorsal end of prelimbic cortex that borders with anterior cingulate cortex (top enlarged image in the middle) to the ventral end of prelimbic cortex that borders with infralimbic cortex (bottom enlarged image in the middle). Varicosities along one axon that is located at the edge of anterior cingulate and prelimbic cortex are shown in a high magnification image (enlarged image on the right; yellow arrowheads mark varicosities). Cell nuclei in the section were counterstained with DAPI (blue). **c** In maximum intensity projections taken over an image stack with 10 single focal plane images that each spanned 0.58 µm ventral motor thalamic axon terminals (green) overlapped with VGluT2 (magenta) and to less extent VGluT1 (magenta) and GAD67 (magenta). VGluT2, VGluT1 and GAD67 were labeled in adjacent slices and, hence, are all shown in magenta. **d** However, in single focal plane images spanning 0.58 µm ventral motor thalamic axon terminals (green) were only found to express VGluT2 (magenta) and to less extent VGluT1 (magenta), but not GAD67 (magenta). Yellow arrowheads in **c** and **d** mark expression of VGluT2, VGluT1 and GAD67 and the yellow dashed lines mark midline

Data on the suitability of the used RGS14 and 5HT3aR antibody for our application (data presented in Figs. 3c, 4 and 5) and data on VGluT2, VGluT1 and GAD67 expression in VM axon terminals in prelimbic cortex (data presented in Fig. 6) are available on figshare (https://dx.doi.org/10.6084/m9.figshare.7039376). Any other data will be made available upon request.

## Results

First, we verified that antibodies for RGS14 and 5HT3aR were suitable to label postsynaptic structures and were labeling CS neurons and layer I inhibitory interneurons, respectively. Such confirmation was of great importance given that both antibodies label membrane proteins. Hence, immunohistochemistry with these antibodies does not label the entire somata or entire dendrites but is seen as a small localized dot pattern. Nevertheless, since RGS14 (Squires et al. 2018) as well as 5HT3aR are primarily found at synaptic sites (Huang et al. 2004), they are still suitable to detect potential contacts between incoming VM axon terminals and postsynaptic structures, specifically if both antibodies are verified to label postsynaptic sites. Because antibodies were predicted to label somata and postsynaptic sites along dendrites, a high background was expected in cortical layers that contain many neurons or their dendrites that are immunoreactive to the antibody.

### RGS14 and 5HT3aR are expressed postsynaptic and are specific

In prelimbic cortical layer I, RGS14 as well as 5HT3aR are expressed at postsynaptic rather than presynaptic sites. Immunohistochemistry for RGS14, SOP and PSD95 or 5HT3aR, SOP and PSD95 revealed that both markers, RGS14 (Fig. 3a) and 5HT3aR (Fig. 3b), are expressed in postsynaptic sites labeled with PSD95 rather than in presynaptic sites labeled with SOP. Hence, both antibodies are suitable to detect contacts between incoming VM axons in prelimbic cortical layer I and postsynaptic structures.

RGS14 is expressed along dendrites of pyramidal neurons (Fig. 3c). RGS14 was suggested as a marker for CS neurons in prelimbic cortex. CS neurons are a specific type of pyramidal neuron. Thus, we used immunohistochemistry to confirm that in prelimbic cortex RGS14 was expressed along dendrites of pyramidal neurons. RGS14 expression is clearly visible along dendrites of pyramidal neurons marked with MAP2 (Fig. 3c).

To verify that somatic and dendritic expression of RGS14 in rat prelimbic cortex is mostly restricted to CS neurons, we retrogradely labeled CS neurons (Fig. 4a, d) or CT neurons (Fig. 4e) in prelimbic cortex by placing injections of the retrograde tracer choleratoxin subunit B in dorsomedial striatum (Fig. 4b) or in the ventral motor and adjacent thalamic nuclei (Fig. 4c). Expression of RGS14 in somata of retrogradely labeled CS neurons was used as a positive control, while absence of expression in somata of retrogradely labeled CT neurons was used as a negative control. While RGS14 was expressed in the nuclei of most pyramidal neurons, in prelimbic cortex somatic expression of RGS14 was mostly restricted to CS neurons (Fig. 4d). Some prelimbic CT neurons that were retrogradely labeled from ventral motor and adjacent thalamic nuclei sparsely expressed RGS14 along the outer membrane of the cell nuclei (Fig. 4e). Furthermore, in very rare cases extremely sparse and weak expression of RGS14 was observed in the somata, but the majority of prelimbic CT neurons did not show any somatic expression of RGS14 (Fig. 4e). Somatic expression in these rare cases may be due to different factors that are discussed below. Specificity for CS neurons is illustrated in three animals (Fig. 4d) and for CT neurons in two animals (Fig. 4e), but data from seven animals and four animals are available on figshare (https://dx.doi.org/10.6084/m9.figshare.7039376).

To verify that 5HT3aR labels the majority of layer I inhibitory interneurons in rat prelimbic cortex, we labeled layer I inhibitory interneurons with GAD67, a neurochemical marker for most inhibitory interneurons (Retaux et al. 1993), and confirmed that all GAD67-positive neurons in rat prelimbic layer I also express 5HT3aR (Fig. 5). Neuronal cells in rat prelimbic layer I that expressed GAD67 in their somata also expressed 5HT3aR, while neurons that did not express GAD67 in their somata also failed to express 5HT3aR (Fig. 5b). Specificity is herein illustrated in three animals (Fig. 5b), but raw data from six animals are available on figshare (https://dx.doi.org/10.6084/m9.figshare.7039376).

Reportedly, double bouquet cells in deeper layers that send dendrites to cortical layer I can also express 5HT3aR (Rudy et al. 2011; Kubota 2014). However, because we only looked at somata to confirm that no false-negative or false-positive staining occurred, 5HT3aR expression in dendrites from deeper layers does not affect validity of these results. Nevertheless, we would like to mention that the estimated contacts onto 5HT3aR-positive inhibitory interneurons in layer I may include contacts onto dendrites of double bouquet cells that are located in deeper layers. To acknowledge this limitation, we will refer to them as contacts onto dendrites of 5HT3aR-positive inhibitory interneurons in layer I from here on.

### Ventral motor thalamic axon terminals in prelimbic cortex are glutamatergic

Ventral motor thalamic axon terminals in prelimbic cortical layer I express VGluT2 and to less extent VGluT1 but not GAD67. To gain a better understanding of the properties of ventral motor thalamic axon terminals, we aimed to verify that their neurochemical properties resemble those of other thalamocortical axon terminals. Injections of AAV5-CAG-ArchT-GFP or AAV5-CAG-GFP that were placed into ventral motor thalamic nuclei (Fig. 6a) verified that the axon terminals spanned the entire length of medial prefrontal cortex and are present in anterior cingulate cortex, prelimbic cortex and orbitofrontal cortex (Fig. 6b). A high magnification image of one axon found in upper prelimbic cortex and varicosities along this axon is provided in Fig. 6b. AAV5-CAG-ArchT-GFP and AAV5-CAG-GFP have previously been used to express archaerhodopsin and green fluorescent protein in axon terminals (Ozawa et al. 2017). While retrograde transport has been observed using AAV5 (Burger et al. 2004), we did not observe any retrograde labeling in prelimbic cortex in this study. The observed labeling pattern resembled the pattern observed in cortex in previous studies that performed anterograde tracing of ventromedial thalamocortical axons with the anterograde tracer *Phaseolus vulgaris* leucoagglutinin (Arbuthnott et al. 1990). Using immunohistochemistry, we corroborated in maximum intensity projections (Fig. 6c) and single focal plane images spanning 0.58 µm (Fig. 6d) that thalamocortical axon terminals mainly express VGluT2 and to a lesser extent VGluT1. Surprisingly, in maximum intensity projections we also observed an overlap of ventral motor thalamic axon terminals and GAD67 expression (Fig. 6c). However, these results were not confirmed in single focal plane images. GAD67 did not superimpose with ventral motor thalamic axon terminals but was only expressed in close proximity (Fig. 6d), providing first evidence that ventral motor thalamic axon terminals may make contacts on inhibitory interneurons that are located in prelimbic layer I and known to express GAD67 (Retaux et al. 1993). Expression is illustrated in one animal (Fig. 6), but raw data from five animals are available on figshare (https://dx.doi.org/10.6084/m9.figshare.7039376).

### VM projections target pyramidal neurons, corticostriatal neurons and layer I inhibitory interneurons

Information on the innervation pattern of VM projection neurons in prelimbic cortex will allow us to make predictions on their connectivity. Thus, we aimed to determine if in the uppermost 40 µm of prelimbic cortical layer I VM axon terminals make contacts onto dendrites of pyramidal neurons, CS neurons and 5HT3aR-positive inhibitory interneurons. A combination of anterograde tracing, immunohistochemistry and light microscopy revealed that in the uppermost 40 µm of prelimbic cortical layer I VM axon terminals indeed make contacts onto dendrites of pyramidal neurons, CS neurons and 5HT3aR-positive inhibitory interneurons (Fig. 9). 3D-reconstruction of selected contact sites further revealed that these are most likely contacts between a presynaptic axon terminal and a postsynaptic site (Fig. 9d).

**Fig. 7:**
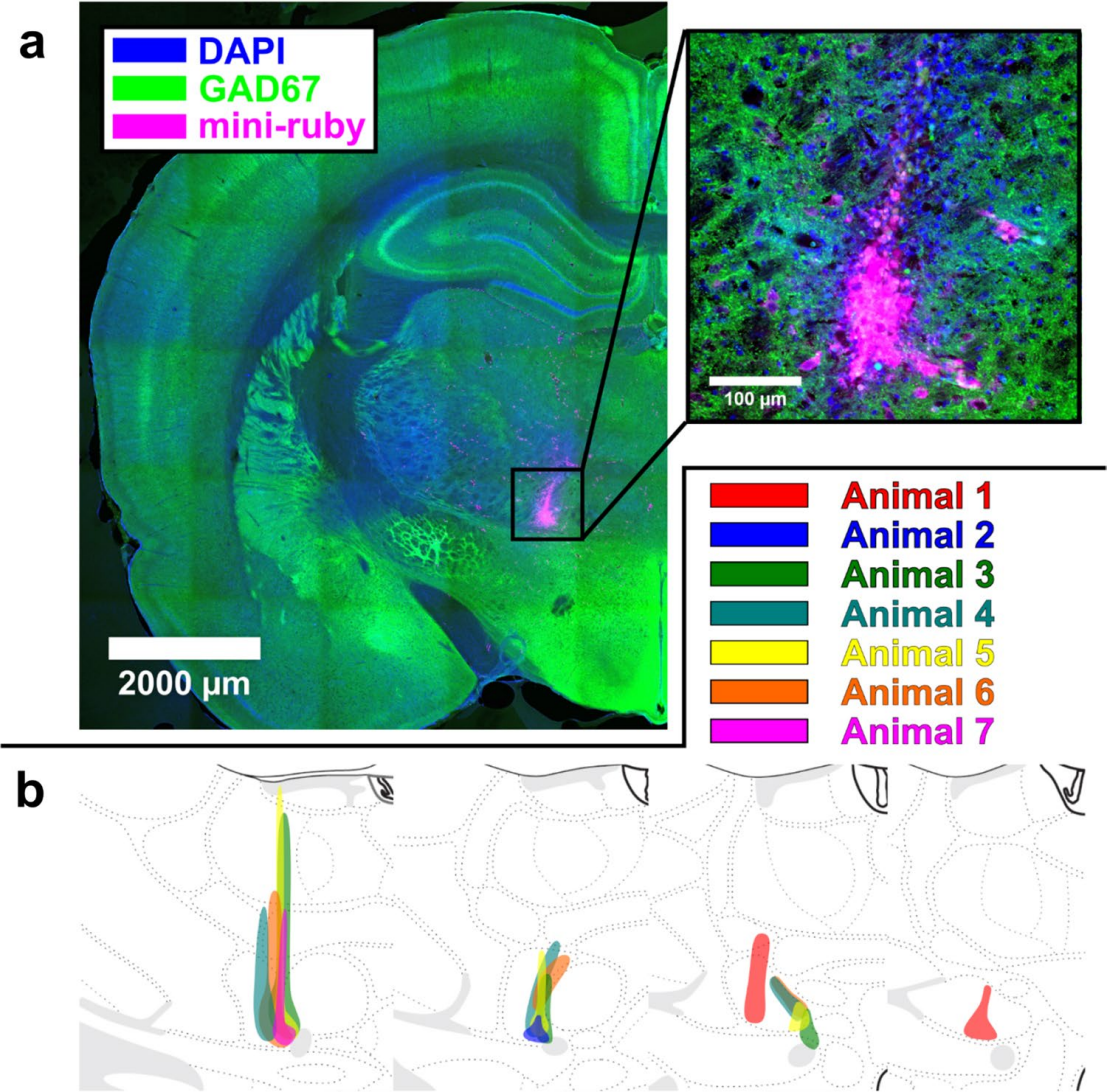
Mini-ruby injections were mostly confined to VM. **a** The anterograde tracer mini-ruby (magenta) was injected into VM that was labeled with GAD67 (green) and counterstained with DAPI (blue) to visualize cell nuclei. **b** The spread of injections is illustrated for all seven animals. Injections were mostly confined to VM. Illustrations of VM and surrounding brain areas are replicated from Swanson (2004), an open-source rat brain atlas

To label VM axon terminals in prelimbic cortex, mini-ruby was injected into VM (Fig. 7a). Injections were mostly confined to VM with minor expression along the needle track in five out of seven animals, and minor spillage into the mammillothalamic tract and submedius thalamic nucleus in two out of seven animals (Fig. 7b). While location of VM was defined in accordance with histological markers and boundaries presented in a rat brain atlas (Paxinos and Watson 2004), injection sites were mapped out on an open-source rat brain atlas (Swanson 2004) to avoid violation of copyright protecting the work of Paxinos and Watson (2004).

**Fig. 8:**
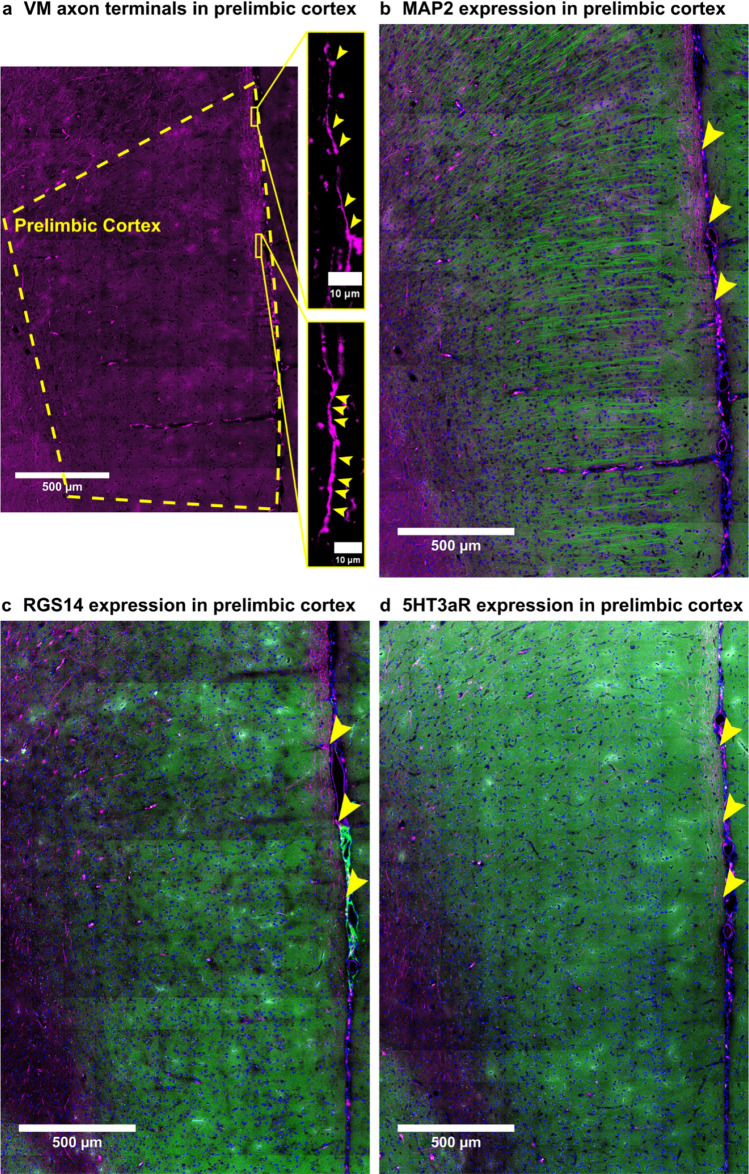
Mini-ruby, MAP2, RGS14 and 5HT3aR expression in prelimbic cortex. **a** The anterograde tracer mini-ruby (magenta) labeled VM axon terminals in prelimbic cortex, which is outlined by a yellow dashed line. High magnification images on the right illustrate that mini-ruby is expressed in axons and varicosities along these axons. Axons and varicosities are marked by yellow arrowheads. **b** MAP2 (green), **c** RGS14 (green) and **d** 5HT3aR (green) are expressed throughout prelimbic cortex. The same section is presented in **a**, **b** with **a** only showing fluorescence from the mini-ruby (magenta), while **b** shows fluorescence from the mini-ruby (magenta), MAP2 (green) and DAPI (blue). In **c** and **d** adjacent sections are shown that also contain fluorescence from mini-ruby (magenta) and were stained for RGS14 (green) and 5HT3aR (green), respectively. Sections have been counterstained with DAPI (blue) to visualize cell nuclei. Yellow arrowheads mark sites imaged to obtain data presented in Fig. 9

**Fig. 9:**
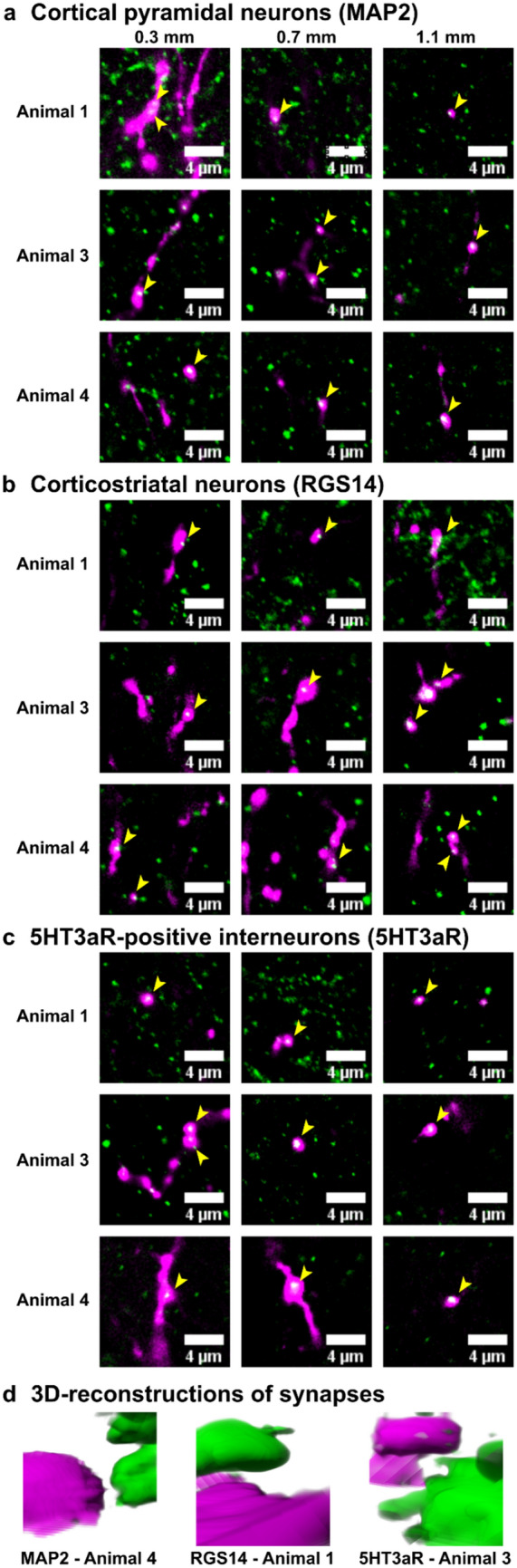
VM axon terminals in prelimbic cortex make contacts onto dendrites of pyramidal neurons, CS neurons and 5HT3aR-positive inhibitory interneurons in layer I. **a** Imaging data were obtained in three animals at 0.3, 0.7 and 1.1 mm ventral from the dorsal end of prelimbic cortex at sites marked with yellow arrowheads in Fig. 8. VM axon terminals in uppermost prelimbic cortical layer I (magenta) superimpose with MAP2 (green) that labels dendrites of cortical pyramidal neurons, **b** RGS14 (green) that labels synaptic sites along dendrites of CS neurons, and **c** 5HT3aR (green) that labels synaptic sites along the dendrites of 5HT3aR-positive inhibitory interneurons. Yellow arrowheads indicate superimpositions. Images illustrating superimpositions have been cropped from single focal plane images. Each single focal plane image spanned 0.29 μm. Animal numbers correspond to animal numbers in Fig. 7, which illustrates the spread of injections in all seven animals. **d** 3D-reconstructions of selected contacts between incoming VM axon terminals and each marker

Expression of mini-ruby in VM axons in prelimbic cortex was confirmed in high resolution images and expression is illustrated by two high magnification images in Fig. 8a. The neuronal markers MAP2, RGS14 and 5HT3aR were expressed throughout prelimbic cortex as illustrated in Fig. 8b–d. VM axon terminals in uppermost prelimbic cortical layer I superimpose with all three neuronal markers (Fig. 9), indicating that in prelimbic cortical layer I VM projection neurons make contacts onto dendrites of pyramidal neurons, CS neurons and 5HT3aR-positive inhibitory interneurons in layer I.

### VM preferentially contacts corticostriatal neurons

**Fig. 10:**
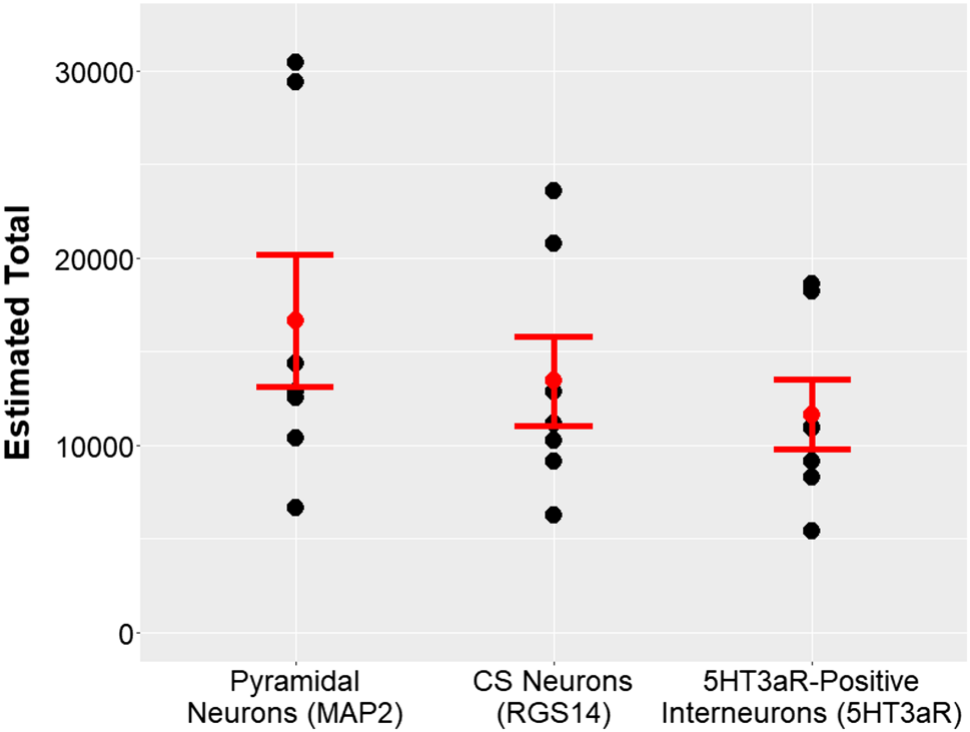
We used systematic random sampling to estimate the total number of contacts between VM axon terminals in the uppermost 40 μm of prelimbic cortex and dendrites of pyramidal neurons marked with MAP2, dendrites of CS neurons marked with RGS14 and dendrites of 5HT3aR-positive inhibitory interneurons in layer I. The estimated number of contacts for each of the seven animals are indicated by black dots. The mean and standard error of the mean for the estimated total amount of contacts are indicated in red

To determine the magnitude of VM contacts onto prelimbic CS neurons as well as onto layer I inhibitory interneurons in comparison to those onto pyramidal neurons, we estimated the total number of contacts in the uppermost 40 µm of prelimbic cortical layer I of VM axon terminals onto dendrites of (1) pyramidal neurons, (2) CS neurons, and (3) 5HT3aR-positive inhibitory interneurons in layer I using systematic random sampling. Mean and the standard error of the mean of the estimated total amount of superimpositions (Fig. 10) was derived from the estimated totals shown in Table 3. About 80% of contacts onto dendrites of pyramidal neuron are made onto dendrites of CS neurons. For every 10 contacts of VM projection neurons onto dendrites of pyramidal neurons about seven contacts are made onto dendrites of 5HT3aR-positive inhibitory interneurons in layer I, indicating that overall VM makes more contacts onto the population of prelimbic pyramidal neurons as compared to that of layer I inhibitory interneurons. Non-parametric Wilcoxon signed-rank tests confirmed that significantly more contacts are made onto dendrites of cortical pyramidal neurons as compared to dendrites of CS neurons (*p* = 0.016, *r* = 0.91) or 5HT3aR-positive inhibitory interneurons in layer I (*p* = 0.016, *r* = 0.91).

**Table 3:** Estimated total amount of contacts for all seven animals between ventromedial thalamic axon terminals in uppermost prelimbic cortical layer I and dendrites of pyramidal neurons marked with MAP2, dendrites of CS neurons marked with RGS14 and dendrites of 5HT3aR-positive inhibitory interneurons in layer I

	Cortical pyramidal neurons (MAP2)	CS neurons (RGS14)	Layer I inhibitory interneurons (5HT3aR)
Animal 1	10,391	9108	8284
Animal 2	12,896	12,880	10,878
Animal 3	12,530	11,175	10,980
Animal 4	14,376	10,225	9120
Animal 5	29,393	23,566	18,251
Animal 6	6650	6270	5413
Animal 7	30,477	20,757	18,624
Mean	16,673	13,426	11,650

The mean is given in the bottom row

## Discussion

First, we verified that in prelimbic cortex somatic expression of RGS14 is present in and mostly limited to CS neurons, even though in very rare cases sparse and weak somatic expression was observed in retrogradely labeled CT neurons. Such expression may have multiple reasons. The retrograde tracer may have leaked into cortex along the injection track and been taken up by axon terminals of some CS neurons; or perhaps expression may originate from dendrites of CS neurons that run in close proximity to CT neurons and express RGS14. Both explanations are plausible given that the specificity of the antibody has been tested by the supplier using immunoblot and immunohistochemistry, and that in prelimbic cortex somatic and dendritic expression of RGS14 has previously been reported to be limited to intratelencephalic CS neurons (Gerfen et al. 2013). Hence, we believe that our data support previous observations that in prelimbic cortex RGS14 can be used as a marker for the somata and dendrites of CS neurons, despite the sparse and weak expression seen along the somata of a limited number of CT neurons.

Next, we marked inhibitory interneurons in cortical layer I with GAD67 and verified that 5HT3aR expression was restricted to them. In rats, 90% of 5HT3aR-positive neurons in cortex and hippocampus are GABAergic (Morales et al. 1996). In mice, 99% of inhibitory interneurons in cortical layer I are 5HT3aR-positive (Rudy et al. 2011; Feldmeyer et al. 2018). In rats, more 5HT3aR-positive cells are found in cortical layers II/III as compared to cortical layer I (Morales et al. 1998), raising doubts that results in mice are translatable to rats. However, in situ hybridization studies showed that expression levels of 5HT3aR in different cortical layers are comparable between rats and mice (Tecott et al. 1993; Morales and Bloom 1997). Thus, a large abundance of 5HT3aR-positive neurons in cortical layers II/III as compared to cortical layer I may simply be due to a generally denser population and a larger number of neurons in cortical layers II/III as compared to cortical layer I. Thus, we believe that the result that 99% of inhibitory interneurons in cortical layer I are 5HT3aR-positive are translatable to rats. Furthermore, when (Morales et al. 2004) tested if 5HT3aR-positive neurons in cortex co-express the cannabinoid CB1 receptor, none of the 5HT3aR-positive inhibitory interneurons in cortical layer I co-expressed the receptor. This result further supports our belief, since most layer I inhibitory interneurons appear to primarily express 5HT3aR. We believe that our results confirm that the used 5HT3aR antibody is suitable to mark prelimbic layer I inhibitory interneurons. However, the antibody may also mark dendrites of 5HT3aR-positive double bouquet cells that are located in deeper layers, but send dendrites to cortical layer I (Rudy et al. 2011; Kubota 2014). Nevertheless, 5HT3aR is the most suitable marker available to label somata and dendrites of layer I inhibitory interneurons, because the alternative marker GAD67 that labels all inhibitory interneurons is only expressed in somata and presynaptic elements, but not in postsynaptic elements along dendrites of layer I inhibitory interneurons. Thus, only staining with 5HT3aR will reveal contacts of incoming axons onto dendrites of layer I inhibitory interneurons.

Finally, we demonstrated that VM axon terminals in the uppermost 40 µm of prelimbic layer I superimpose with MAP2, RGS14 and 5HT3aR. This result suggests that VM projection neurons in uppermost prelimbic cortical layer I make contacts onto dendrites of pyramidal neurons marked with MAP2, onto dendrites of CS neurons marked with RGS14 and onto dendrites of 5HT3aR-positive inhibitory interneurons in layer I. Previous evidence that VM projections target layer I inhibitory interneurons in medial prefrontal cortex has been sparse. Cruikshank et al. (2012) provided evidence that optical stimulation of midline thalamic axon terminals in secondary motor cortex and anterior cingulate cortex can evoke a response in layer I inhibitory interneurons. Some injections of channelrhodopsin, the compound that was used to induce optical stimulation in midline thalamic axon terminals, were placed in VM. However, the extent of the injections was not reported in the article. Our results extend this evidence using anatomical methods and clearly indicate that VM projection neurons to prelimbic cortex make contacts onto dendrites of layer I inhibitory interneurons in rats. Since layer I inhibitory interneurons can induce feedforward inhibition in pyramidal neurons (Jiang et al. 2013, 2015), this result indicates that VM input to prelimbic cortex may not only induce excitatory responses in prelimbic pyramidal neurons but can also inhibit them. Feedforward inhibition through VM input is not limited to prelimbic pyramidal neurons innervated by VM axon terminals. VM input may further induce feedforward inhibition in prelimbic pyramidal neurons that receive input from other thalamic or cortical regions, i.e., mediodorsal thalamus, reuniens thalamus or contralateral orbitofrontal cortex. Moreover, the presented results provide anatomical confirmation of previous results that were based on *in-vitro* electrophysiology and demonstrated that VM projections induce strong excitatory postsynaptic currents in intratelencephalic neurons (Collins et al. 2018).

In general, the resolution of light microscopy makes it challenging to visualize axon terminals or to localize small proteins, but a more recent study reported that visualization of synaptic contacts of axon terminals onto cell bodies with a state-of-the-art confocal microscope yielded the same results as visualization with electron microscopy (Matsuno et al. 2016). Older studies reported that not all contacts between axon terminals and cell bodies that were observed with confocal microscopy were confirmed to be synaptic contacts with electron microscopy (Jankowska et al. 1997; Cabirol-Pol et al. 2000). However, even in these two studies at least a subset of contacts was identified as synaptic contacts using electron microscopy. Hence, the number of superimpositions or contacts observed in this study using state-of-the-art confocal microscopy most likely correlates with the number of synaptic contacts. If we overestimated the number of contacts, the factor that we overestimated by should be consistent across estimates. Thus, we believe that the presented ratios between estimated contacts still reflect ratios of synaptic contacts.

Resolution of most of the obtained image stacks could have been further improved by applying deconvolution, specifically adaptive blind deconvolution, a technique that can decrease blurring in images obtained with confocal microscopy (Biggs 2010a, b). However, we decided not to apply deconvolution due to two reasons: (1) the cited journal articles, which showed that at least a subset of contacts found with state-of-the-art confocal microscopy is also visible in electron microscopy, did not apply deconvolution to their confocal images (Jankowska et al. 1997; Cabirol-Pol et al. 2000; Matsuno et al. 2016) and we did not want to deviate too far from techniques applied for data analysis in these articles. (2) To apply adaptive blind deconvolution to an image stack, stepping distance must fulfill the Nyquist theorem (Biggs 2010a, b). Thus, adaptive blind deconvolution could not be applied to the image stacks that were used to quantify contacts using systematic random sampling due to the large stepping distance in *z*-direction between single focal plane images in these stacks. However, due to the large area that we needed to cover in each slice, and technical limitations of the available equipment, we were unable to use a smaller stepping distance. Since we wanted to provide a realistic image to readers that depicts the nature of contacts counted during the quantification process, we aimed to process all images in a comparable way. Hence, no deconvolution was applied to any of the presented image stacks. Nevertheless, contacts defined as a superimposition of at least four voxels into *x*- and *y*-direction were clearly visible in many of the acquired image stacks.

Our results are further limited, because we cannot confirm with complete certainty whether RGS14, 5HT3aR and VGluT1 are expressed in presynaptic terminals of incoming VM/ventral motor thalamic axon terminals or at postsynaptic sites. MAP2 expression is clearly limited to dendrites of pyramidal neurons (Bernhardt and Matus 1984), but RGS14 and 5HT3aR could be expressed in presynaptic terminals of incoming VM axon terminals rather than in postsynaptic terminals located along dendrites of CS neurons or layer I inhibitory interneurons. However, the current results demonstrate that in prelimbic cortical layer I RGS14 and 5HT3aR are most likely expressed at postsynaptic and not presynaptic sites. Besides, previous studies that used transgenic mice did not report any presynaptic expression of RGS14 and 5HT3aR along incoming axon terminals in cortical layer I (Rudy et al. 2011; Gerfen et al. 2013). Moreover, while VGluT2 expression in cortex is limited to thalamocortical axon terminals (Fujiyama et al. 2001; Kaneko and Fujiyama 2002; Hur and Zaborszky 2005), VGluT1 could be expressed in other postsynaptic terminals, e.g., of cortical pyramidal neurons (Fremeau et al. 2001), and mark superimpositions between incoming VM axon terminals and cortical pyramidal neurons.

Since we applied quantitative anatomical methods, we were able to estimate the approximate number of contacts between VM axon terminals located in the uppermost 40-µm of prelimbic cortical layer I and dendrites of pyramidal neurons, CS neurons or 5HT3aR-positive inhibitory interneurons in layer I. For every seven contacts made onto dendrites of layer I inhibitory interneurons, 10 contacts are made onto the dendritic tuft of pyramidal neurons, indicating that overall in prelimbic cortex VM makes more contacts on the population of excitatory pyramidal neurons than on that of layer I inhibitory interneurons. Thalamic input to cortical inhibitory interneurons, specifically from posterior medial thalamic complex to primary somatosensory cortex, was recently suggested to be critical for the induction of long-term depression in cortical pyramidal neurons (Williams and Holtmaat 2019). Given the extensive input to layer I inhibitory interneurons, VM input to prelimbic cortex may play a role in similar mechanisms and as such may be implicated in memory formation or learning.

Results further imply that about 80% of VM axon terminal contacts onto dendrites of cortical pyramidal neurons are made onto dendrites of CS neurons, a specific kind of pyramidal neurons. Innervation patterns of VM projection neurons and specifically the kind of pyramidal neurons that are targeted reportedly differ across cortical areas, e.g., VM projection neurons to somatosensory cortex target corticocortical, corticothalamic and pyramidal tract neurons (Thomson 2010), while those in motor cortex only target corticocortical and pyramidal tract neurons (Yamawaki and Shepherd 2015). Previous results showed that optical stimulation of VM axon terminals in prelimbic cortex evokes strong responses in layers II/III and layer V corticocortical neurons, which project to contralateral cortex and striatum, and weaker responses in layer V corticothalamic neurons (Collins et al. 2018). The current results extend these findings and provide further insight into the innervation pattern of VM projection neurons in prelimbic cortex demonstrating that those pyramidal neurons targeted most by VM projection neurons are CS neurons. In contrast, VM projection neurons to ALM, a different prefrontal cortical area, provide input to layer VB pyramidal tract and weaker input to layers II/III corticocortical neurons, but not to identified corticothalamic neurons in layer VI (Guo et al. 2018). Given such dramatic differences in VM innervation patterns even within prefrontal cortical areas, the current results provide important insights for further studies. In addition, because prelimbic CS neurons are involved in cost–benefit decision-making (Friedman et al. 2015, 2017), the current results provide neuroanatomical evidence for an involvement of ventral motor thalamic input to prelimbic cortex in cost–benefit decision-making.
